# Differential heterologous neutralisation profile against strains within DENV-3 genotype II

**DOI:** 10.1017/S0950268821002648

**Published:** 2021-12-17

**Authors:** K.K. Tan, S. Abubakar

**Affiliations:** 1 Tropical Infectious Diseases Research and Education Centre (TIDREC), Higher Institution Centre of Excellence (HICoE), Universiti Malaya, 50603 Kuala Lumpur, Malaysia; 2 Department of Medical Microbiology, Faculty of Medicine, Universiti Malaya, 50603 Kuala Lumpur, Malaysia

**Keywords:** Dengue virus, immunity, infectious diseases, tropical, vector

## Abstract

The dengue virus type 3 (DENV-3) homotypic outbreak cycles reported in Klang Valley, Malaysia in 1992–1995 and 2002 demonstrated different epidemic magnitude and duration. These outbreak cycles were caused by two closely related strains of viruses within the DENV-3 genotype II (DENV-3/II). The role of viral genotypic diversity and factors that could have influenced this phenomenon were investigated. The serum neutralisation sensitivity of DEN3/II strains responsible for the DENV-3 outbreak cycles in 1992–1995 and 2002 were examined. Representative virus isolates from the respective outbreaks were subjected to virus neutralisation assay using identified sera of patients with homotypic (DENV-3) or heterotypic dengue infections (DENV-1 and DENV-2). Results from the study suggested that isolates representing DENV-3/II group E (DENV-3/II-E) from the 1992–1995 outbreak and DENV-3/II group F (DENV-3/II-F) from the 2002 outbreak were neutralised at similar capacity (intergenotypic differences <2-fold) by sera of patients infected with DENV-3, DENV-1 and DENV-2/Asian genotypes. Sera of the DENV-2/Cosmopolitan infection efficiently neutralised DENV-3/II-F (FRNT_50_ = 508.0) at a similar neutralisation capacity against its own homotypic serotype, DENV-2 (FRNT_50_ = 452.5), but not against DENV-3/II-E (FRNT_50_ = 100.8). The different neutralisation sensitivities of DENV-3/II strains towards the cross-reacting DENV-2 heterotypic immunity could play a role in shaping the DENV-3 recurring outbreaks pattern in Malaysia. Two genetic variations, E-132 (H/Y) and E-479 (A/V) were identified on the envelope protein of DENV-3/II-E and DENV-3/II-F, respectively. The E-132 variation was predicted to affect the protein stability. A more extensive study, however, on the implication of the naturally occurring genetic variations within closely related DENV genotypes on the neutralisation profile and protective immunity would be needed for a better understanding of the DENV spread pattern in a hyperendemic setting.

Dengue is a mosquito-borne disease affecting over half of the world's population. It is over a century old disease in Malaysia [1]. All four dengue virus (DENV) serotypes caused dengue and the different DENV serotypes have co-circulated in many regions of the world. In the Klang Valley, Malaysia, the major outbreak cycles involving the same DENV serotype, the homotypic cycle, occurred at every 4–10 years interval [2, 3]. The major outbreak cycles involving the different serotypes, the heterotypic cycle, occurred in a sequential manner involving DENV-3, followed by DENV-1, and then DENV-2 (DENV-3/DENV-1/DENV-2) in that order [2]. So far, only three DENV-3/DENV-1/DENV-2 supra-serotype cycles have been recorded [2]. The DENV-4 maintained low circulation in the background of DENV-1, DENV-2 and DENV-3 [2]. DENV-3-homotypic cycles were recorded in 1986, 1992–1995 and 2002 [2]. The anticipated DENV-3 homotypic cycle, which could appear between 2006 and 2012, did not materialise [2]. Notably, during the 2002 DENV-3 homotypic cycle, DENV-3 was only implicated in 42% of the total serotyped cases compared to the 1992–1995 cycle, where DENV-3 contributed up to 90% of the typed DENV cases [2]. Although several DENV-3 genotypes have been found in Malaysia for the past-30 years [2, 4, 5], DENV-3 genotype II (DENV-3/II) has been the only genotype associated with the outbreak cycles [2]. Previous genetic analysis showed that these cycles were caused by two closely related groups within DENV-3/II [6]. The different outbreak magnitude and duration of DENV-3/II-associated homotypic outbreak cycles in the same locality suggests possible underlying factor(s) contributing to the differences. Immune-driven selection of virus clade that can escape host immunity has been shown as one of the possible mechanisms for selecting the dominant circulating strains of many infectious agents [4, 7] and shaping the epidemic pattern [8]. To understand if closely related strains within the same DENV-3 genotype would respond similarly to the existing DENV immunity in the local setting, we investigated the neutralisation capacities of homotypic and heterotypic immune sera against the representative DENV-3/II strains of 1992–1995 and 2002 DENV-3 outbreaks. The serum sample against DENV-4 infection was not available; hence, the heterotypic neutralisation by DENV4 serum was not assessed in the current study. Subsequently, we examined the genetic variations between the DENV-3/II strains that could influence the neutralisation capacity.

The representative DENV-3 strains responsible for 1992–1995 (DENV-3/II-E) and 2002 (DENV-3/II-F) outbreaks were prepared by serial propagation of the selected DENV-3 strains in C6/36 mosquito cells (*Aedes albopictus* cell line). The use of human serum samples in this study was approved by the Medical Ethics Committee of the University Malaya Medical Centre (MEC Ref No: 806.23 and 806.24). The dengue status of selected human serum samples was characterised using the anti-dengue IgM and IgG as previously described [4]. The IgM/IgG ratio of more than 1.4 was classified as the primary infection. The patients' sera with IgM/IgG ratio less than 1.2 or the detection of anti-dengue IgG concurrent with the isolation of DENV was characterised as a secondary infection [9]. The serum samples with IgM/IgG ratios between 1.2 and 1.4 were excluded from this study. The serum from donors tested negative for DENV antibodies (IgG and IgM) were used as a negative control. The selected serum samples were used in the focus reduction neutralisation test (FRNT) against the different DENV-3 strains as previously described [4]. The patients' sera representing the homotypic and heterotypic immunity in the local population used in the current study were listed in Table 1.

**Table 1.** List of patients' sera used in this study

No	Patients' sera	Infecting DENV	Circulating period
1	sDENV-1/I	DENV-1 genotype I	Predominant circulating DENV-1 during 1987, 1997 and 2004 DENV-1 recurring outbreaks [16].
2	sDENV-1/II	DENV-1 genotype II	Circulating DENV-1 during the 1987 and 2004 DENV-1 recurring outbreaks [16].
3	sDENV-2/Asian	DENV-2 Asian genotype	The minor circulating DENV-2 serotype in the 1990s.
4	sDENV-2/Cos	DENV-2 Cosmopolitan genotype	Predominant circulating DENV-2 for the past 30 years [13].
5	sDENV-3/I	DENV-3 genotype I	Predominant circulating DENV-3 after 2002 [2].
6	sDENV-3/II-E	DENV-3 genotype II-E	Predominant circulating DENV-3 during 1992–1995 DENV-3 outbreaks.
7	sDENV-3/II-F	DENV-3 genotype II-F	Predominant circulating DENV-3 during 2002 DENV-3 outbreaks.
8	sDENV-3/III	DENV-3 genotype III	DENV-3 genotype emerged in Klang Valley after 2008 [5]

Overall, the control sera (Negative sera) showed weak and non-reactive neutralisation responses against all DENV strains tested (FRNT_50_<40; Table 2). All immune sera (homotypic and heterotypic) demonstrated specific neutralisation capacity (FRNT_50_>40) against tested DENV-3 strains (DENV-3/E and DENV-3/F). The immune sera from patients with secondary infection neutralised DENV-3 strains effectively (FRNT_50_⩾5120). The neutralisation titres of the homotypic immune sera (sDENV-3/I, sDENV-3/II-E, sDENV-3/II-F) against DENV-3/II-E and DENV-3/II-F ranged from 452.5 to 1280. The homotypic serum neutralisation capacity against DENV-3/II-E and DENV-3/II-F were similar with less than 2-fold of intergenotypic differences. For the analysis of heterotypic neutralisation of DENV-1 and DENV-2 immune sera against DENV-3 strains, results showed both DENV-1 immune sera (sDENV-1/I and sDENV-1/II) possess similar neutralisation capacities, with less than 2-fold of intergenotypic differences against DENV-3/II-E and DENV-3/F strains. By using DENV-1 homotypic neutralisation results (sDENV-1 neutralised DENV-1) as control, the sDENV-1/I neutralised DENV-3/II strains more efficiently than sDENV-1/II. The sDENV-1/I neutralised DENV-3/II-E (FRNT_50_ = 640.0) and DENV-3/II-F (FRNT_50_ = 806.3) were less than four-fold differences as compared to neutralisation against DENV-1 (FRNT_50_ = 2031.9). The neutralisation capacity of the sDENV-1/II against DENV-3/II strains (FRNT_50_ = 254.0–320.0), however, differed by at least four-fold to its own DENV-1 strain (FRNT_50_ = 1280). For the heterotypic neutralisation of DENV-2 immune sera, the neutralisation efficiency of the sDENV-2/Cos against DENV-3/II-E (FRNT_50_ = 100.8) was 5.04-fold lesser compared to DENV-3/II-F (FRNT_50_ = 508.0). The DENV-3/II-F was neutralised by sDENV-2/Cos at a similar neutralisation capacity against its own serotype, DENV-2 (FRNT_50_ = 452.5). The neutralisation capacities of sDENV-2/Asian against both DENV-3/II-E and DENV-3/II-F (FRNT_50_ = 160–226.2) were four-fold less efficient than that against DENV-2 homotypic neutralisation (FRNT_50_ = 905.1).

**Table 2.** Neutralisation capacity of DENV immune serum against DENV-3/II strains

Sera	Primary/Secondary	DENV			
		DENV-3/II-F	DENV-3/II-E	DENV-1	DENV-2
Neg sera		25.2	10	15.9	10.0
sDENV-3/I-S	Secondary	⩾5120.0	⩾5120.0	–	–
sDENV-3/III-S	Secondary	⩾5120.0	⩾5120.0	–	–
sDENV-3/I	Primary	452.5	640.0	–	–
sDENV-3/II-E	Primary	1280.0	1280.0	–	–
sDENV-3/II-F	Primary	806.3	640.0	–	–
sDENV-1/I	Primary	806.3	640.0	2031.9	–
sDENV-1/II	Primary	254.0	320.0	1280.0	
sDENV-2/Asian	Primary	160.0	226.2	–	905.1
sDENV-2/Cos	Primary	508.0	100.8	–	452.5

DENV Envelope (E) protein is the main target protein of the neutralising antibodies. We examined the presence of naturally occurring amino acid variations encoded in DENV-3/II-E and DENV-3/II-F E protein. There were two amino acid variation sites, 132 H/Y and 479 V/A in E between the DENV-3/II-E and DENV-3/II-F (Fig. 1). The 132 H/Y is partially accessible residue located in domain I and 479 V/A in the transmembrane region. The solvent accessibility and the potential effects of the amino acid variation on protein stability were predicted using a Site-Directed Mutator (SDM) [10]. The genetic variation at residue E-132 was predicted to affect protein stability (Fig. 2).

**Fig. 1.**
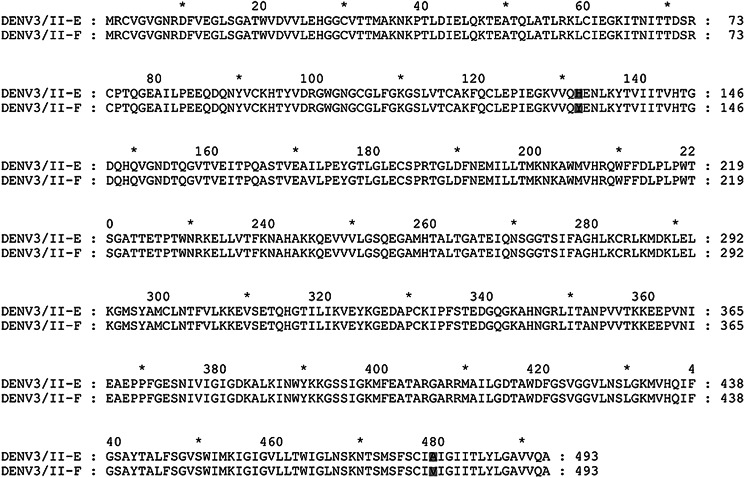
Genetic variations among DENV-3 genotype II within the envelope protein. The variation sites of DENV-3/II-E and DENV-3/II-F were highlighted in grey.

**Fig. 2.**
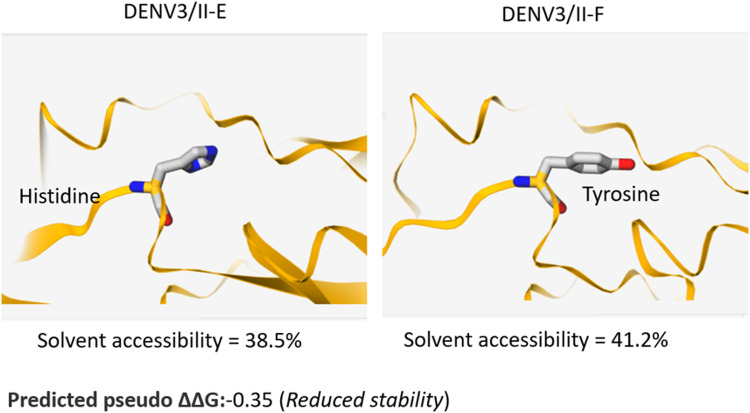
Structural-based prediction of mutation-induced protein stability changes on DENV-3 ectodomain by SDM.

Overall, serum neutralisation results showed that both DENV-3/II-E (1992–1995 DENV-3 cycle) and DENV-3/II-F (2002 DENV-3 cycle) were sensitive to neutralisation by all homotypic (DENV-3) and heterotypic DENV-1 and DENV-2 sera. Our findings suggest that the broad cross-neutralisation of DENV-3/II strains by serum from both homotypic and heterotypic DENV serotypes could have mitigated transmission of DENV-3/II strains in DENV endemic areas such as Klang Valley [2, 3]. At any given time, the DENV transmission pattern at that location during the virus introduction could have been influenced by the dengue prevalence, the length of time since the last outbreak and the predominant DENV serotype responsible for the outbreaks. Many previous studies also suggested that the pre-existing population immune landscape and the circulating DENV genotypes act as the determinants that could shape the local virus transmission pattern [4, 11, 12]. Our study showed that different amplitude and outbreak cycles duration were caused by closely related DENV-3/II strains. The differential neutralisation of DENV-3/II genotypes by the cross-neutralising serum from patients with DENV-2 Cosmopolitan infection was observed. The DENV-3/II-F was effectively neutralised at a similar capacity against DENV-2, but not DENV-3/II-E. In Klang Valley, dengue outbreaks occurred in DENV-3/DENV-1/DENV-2 supra-serotype outbreak cycles pattern [2], where the local population have a high level of DENV-2 immunity but low DENV-1 immunity before the emergence of DENV-3 -associated outbreak cycles (Fig. 3). The DENV2 contributed to 54–86% and 57–63% of the dengue cases between 1990–1991 and 2000–2001 (2 years before the 1992 and 2002 outbreaks; Fig. 3). It was noted that the DENV-2/Cosmopolitan genotype was the dominant DENV serotype before the emergence of 1992–1995 and 2002 DENV-3 outbreaks cycles [13]. Hence, the high level of pre-existing DENV-2 immunity before the emergence of DENV-3 was mainly against the DENV-2/Cosmopolitan genotype. The susceptibility of DENV-3/II-F to DENV-2/Cosmopolitan serum could have mitigated its wider transmission, especially in DENV-2 endemic area as compared to DENV-3/II-E, resulting in a short and relatively low epidemic involving DENV-3/II-F reported in 2002. A similar observation was reported in our previous study where the DENV-2 immunity effectively neutralised DENV-3/I but not DENV-3/III [4]. Collectively, the neutralisation disparity of DENV-2 immune sera against different DENV-3 genotypes suggests that the DENV-2 heterotypic immunity could play a role in shaping the DENV-3 recurring outbreaks. The current study is a retrospective study, hence it completely relied on the type and quantity of samples stored in the repository. As only limited volume of serum samples were available, the current study, therefore, is limited to using pooled serum samples in the neutralisation assays

**Fig. 3.**
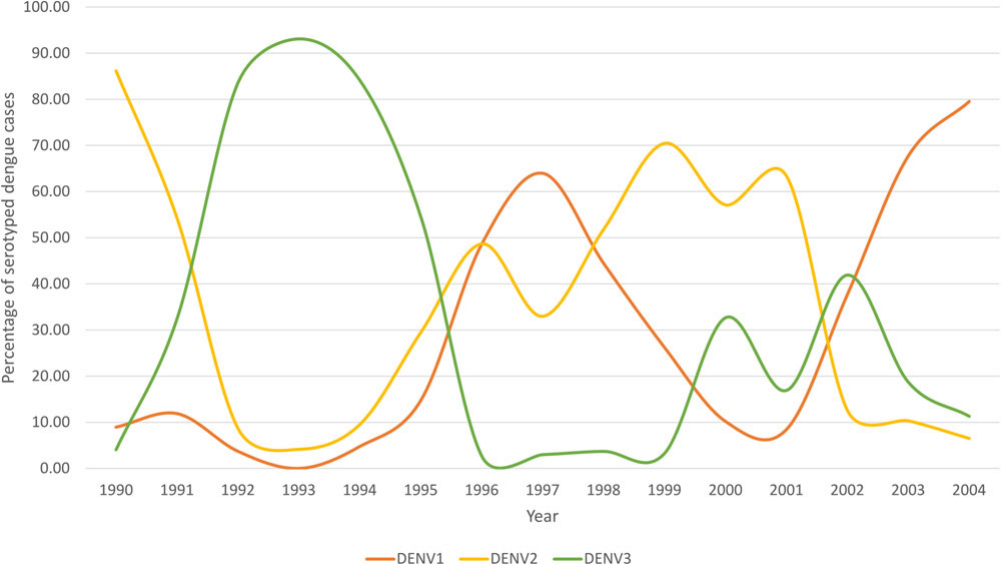
Percentage of DENV-1, DENV-2 and DENV-3 isolated from University Malaya Medical Centre, Klang Valley.

.

Our study also revealed one amino acid difference at position 132 of E protein between DENV-3/E (Histidine) and DENV-3/II-F (Tyrosine). Both histidine and tyrosine are aromatic amino acids commonly found in the semi-buried area [14]. It was noted that the E-132 is a partially accessible residue located at domain I. It was previously identified as a critical residue interacting with the hinge region during conformational changes in fusion trimer formation [15]. Our results suggest that amino acid substitution at E-H132Y could lead to changes in amino acid solvent accessibility. Whether or not these biochemical differences involving histidine and tyrosine at E-132 would influence the contact of the DENV-2/Cosmopolitan antibodies against the DENV-3/II strains that may lead to the differential neutralisation between DENV-3/II-F and DENV-3/II-E remain to be investigated.

In summary, we described the disparity of DENV-2/Cos immune sera neutralisation capacities against DENV-3/II-E and DENV-3/II-F. This differential ability could have contributed to the different virus transmission efficiencies in the DENV-2 endemic areas, the Klang Valley pre-emergence of DENV-3, leading to different outbreak patterns. These findings could be useful to facilitate the understanding of the DENV spread pattern in a hyperendemic setting. Our findings also highlight the need to investigate further how the naturally occurring genetic variations within closely related DENV genotypes could impact the neutralisation profile and protective immunity.

## Data Availability

The datasets used in the current study are available upon request from the corresponding author (SAB).
